# The role of knee arthroscopy in managing common soft tissue complications after total knee arthroplasty: a retrospective case series study

**DOI:** 10.1186/s13018-020-02112-8

**Published:** 2020-12-01

**Authors:** Yunfei Hou, Jiaxiang Gao, Jian Chen, Jianhao Lin, Lei Ni, Tiezheng Sun, Jun Jiang

**Affiliations:** Arthritis Care and Research Center, Peking University People’s Hospital, No.11, Xizhimen South Street, Xicheng District, Beijing, 100044 China

**Keywords:** Knee arthroscopy, Total knee arthroplasty, Peripatellar impingement, Arthrofibrosis, Generalized synovitis, Feasibility

## Abstract

**Background:**

To investigate the feasibility, safety and therapeutic efficacy of arthroscopy in managing the 3 most common soft tissue complications, peripatellar impingement (PI), arthrofibrosis (AF) and generalized synovitis (GS), after total knee arthroplasty (TKA).

**Methods:**

A retrospective review of patients undertaking arthroscopy for PI, AF and GS was conducted. Outcome measures included complications, postoperative range of motion (ROM), Knee Society Score (KSS) and rates of symptom recurrence, prosthesis revision. Intraoperative findings and surgical procedures were also recorded. Paired *t* test, Fisher’s exact test, Kruskal-Wallis test and post hoc analysis with Bonferroni correction were used for statistical evaluation.

**Results:**

A total of 74 patients, including 35 patients with peripatellar impingement, 25 patients with arthrofibrosis and 14 patients with generalized synovitis, with a mean age of 66.1 ± 7.9 years, were analysed. The mean follow-up (FU) duration was 81.3 ± 40.6 months. All patients underwent arthroscopic surgery safely without intraoperative complications. However, there were 4 postoperative complications, including 1 acute myocardial infarction and 3 periprosthetic joint infections. Overall, patients acquired improvements in ROM from 81.7 ± 23.1° to 96.8 ± 20.5° (*p* < 0.05), in KSS knee score from 64.2 ± 9.6 to 78.7 ± 12.1 (*p* < 0.05) and in KSS function score from 61.1 ± 7.4 to 77.3 ± 12.2 (*p* < 0.05) postoperatively. Patients in all 3 groups had improvements in ROM (*p* < 0.05), KSS knee score (*p* < 0.05) and KSS function score (*p* < 0.05). The overall recurrence rate was 22.9% (95% confidence interval (CI) 15.1–34.9%), and the overall revision rate was 14.9% (95% CI 8.6–25.6%). There were significant differences in both the symptom recurrence and prosthesis revision rates among the groups (*p* < 0.05). The PI group had a significantly lower symptom recurrence rate (11.4%, 95% CI 4.5–28.7%) and revision rate (8.6%, 95% CI 2.9–25.3%) (*p* < 0.017), while the GS group had a significantly higher recurrence rate (42.9%, 95% CI 23.4–78.5%) and revision rate (35.7%, 95% CI 17.6–72.1%) (*p* < 0.017).

**Conclusions:**

In the setting of symptomatic TKA, although carrying certain risks for PJI and other complications, arthroscopic intervention could be feasible and provide clinical improvement in most cases at an average of 81.3 months follow-up. Patients with PI had the best outcomes, while patients with GS had the worst outcomes.

**Level of evidence:**

Level IV

## Background

Total knee arthroplasty (TKA) is successful for end-stage knee arthrosis. However, there were approximately 20% dissatisfied patients following primary TKA [1]. Aseptic loosening, instability, patellofemoral (PF) maltracking and periprosthetic joint infection (PJI) are common causes of failure, for which prosthesis revision may be indicated [2]. However, compared to primary surgery, revision TKA is associated with a higher failure rate and compromised outcome [3]. As suggested by a recent systematic review (SR) by Heaven et al. [4], arthroscopy could deal with some of the soft tissue problems after TKA. In the SR, if not including those patients who undertook arthroscopy for diagnostic purposes, the most prevalent three indications were peripatellar impingement (PI), arthrofibrosis (AF) and generalized synovitis (GS) [4].

Arthroscopic surgery is more in line with the principles of contemporary minimally invasive surgery, after which the patient recovers faster and carries a lower risk of infection and other complications theoretically; however, previous research has underreported such issues. Some has not reported this data [5–9] while some reported that there had been no complications [10–16]. Those studies had shortcomings in that they involved relatively few patients, leading to limited statistical power, and the follow-up period was also short. As the follow-up period lengthens, late infection might occur [17]. Lovro et al. noted that knee arthroscopy after arthroplasty was not a benign procedure, with a significantly higher risk of PJI compared to post-TKA patients who did not undergo arthroscopy; however, the authors did not report the conditions of other non-infectious complications [17]. In terms of therapeutic efficacy, the results from previous studies have varied [5, 10, 11]. Thus, the primary objective of the present study was to investigate the feasibility and safety of arthroscopic procedures in patients with soft tissue complications after TKA with longer term of FU, while the secondary objective was to report its therapeutic efficacy separately in the 3 most common indication groups (PI, AF and GS). We hypothesized that arthroscopy is feasible in managing common soft tissue complications after TKA and could provide clinical improvement to varying extents in different indications.

## Methods

### Subjects

Following Institutional Review Board (IRB) approval (IRB approval number 2019 PHB183-01), a retrospective review was performed on 121 consecutive patients (121 knees) who underwent ipsilateral arthroscopy due to uncomfortable TKA from March 2005 to February 2020. The indications for arthroscopy were as follows: (1) patients had a symptomatic TKA and could not recover from conservative treatment, including rest, physiotherapy, braces, medications and/or injections and (2) preoperative history taking, physical exams and laboratory and imaging tests ruled out major abnormalities, such as limb malalignment, joint instability, component malposition, loosening and obvious patellar maltracking, as well as PJI and fracture.

**Fig. 1:**
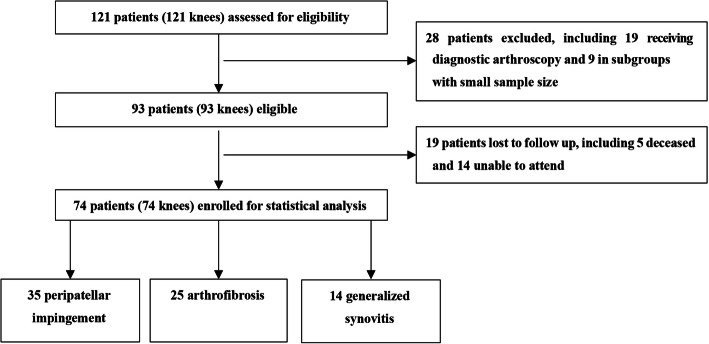
Flow chart of the study. A total of 121 consecutive knee arthroscopy procedures were performed, out of which 93 patients met our inclusion criteria. In all, 74 patients (74 knees) were enrolled for statistical analysis

Exclusion criteria were as follows: (1) patients receiving arthroscopy for diagnostic purposes (*n* = 19), which can be categorized (according to intraoperative findings) into the following conditions: no major pathology identified (*n* = 8), suspected PJI and intraoperative tissue sampling (*n* = 4), loose body identified (*n* = 2), tibial prosthesis loosening (*n* = 2), femoral prosthesis debonding (*n* = 2), dissociation of the tibial metal component from its PE insert (*n* = 1) and (2) patients with known preoperative aetiology but with a sample size that was less than 5, including drainage removal (*n* = 1), popliteal tendon dysfunction and posterolateral corner reconstruction (*n* = 1), concurrent arthroscopy and medial collateral ligament (*n* = 1) or extensor mechanism repair (*n* = 3) and recurrent patellar dislocation (*n* = 3) (the sample size was too small for statistical reporting). In all, 19 patients were lost to FU (5 patients died, 14 patients could not attend the FU). Thus, 74 knees in 74 patients were enrolled for statistical analysis (Fig. 1). Informed consent was obtained from each patient in the current research.

Patient demographics by intraoperative findings are demonstrated in Table 1. In all, 74 patients, including 35 patients with PI, 25 patients with AF and 14 patients with a mean age of 66.1 years were analysed. The original procedure was all TKAs, including 70 posterior stabilized (PS) and 5 cruciate retaining (CR) prostheses. Before arthroscopy, the patients had suffered with symptoms for 11.3 months (range, 2 to 41 months) on average. Patients were followed for 81.3 months, (range, 6 to 118 months). The age and interval between arthroscopy and arthroplasty did not differ among the groups (*p* > 0.05). Preoperatively, patients with AF had significantly less ROM (*p* < 0.003). Patients with GS had a significantly lower KSS knee score (*p* < 0.003) and KSS function score (*p* < 0.003) (Games-Howell post hoc analysis).

**Table 1:** Patient demographics and outcome measures according to indications

	Indications				*p* value
	Overall	PI	AF	GS	
Number of patients/knees	74	35	25	14	
Males to females	8:66	4:31	2:23	2:12	*p* < 0.05
Age (years)	66.1 ± 7.9	66.4 ± 6.2	63.9 ± 9.9	69.4 ± 7.1	0.322
Interval between operations (months)	16.5 ± 14.1	18.4 ± 15.1	15.6 ± 12.5	13.3 ± 14.3	0.381
ROM preop	81.7 ± 23.1	101.3 ± 6.6	56.4 ± 8.3	78.1 ± 21.6	*p* < 0.05
ROM postop	96.8 ± 20.5	110.4 ± 8.5	82.8 ± 16.8	87.9 ± 25.9	*p* < 0.05
Δ ROM	15.1 ± 12.2	9.1 ± 6.5	26.4 ± 12.5	9.8 ± 8.3	*p* < 0.05
KSS knee score preop	64.2 ± 9.6	69.8 ± 3.3	62.7 ± 6.4	52.6 ± 13.5	*p* < 0.05
KSS knee score postop	78.7 ± 12.1	82.3 ± 7.2	78.5 ± 11.7	69.9 ± 17.6	0.235
Δ KSS knee	14.5 ± 9.1	12.5 ± 7.7	15.8 ± 8.7	17.3 ± 12.1	0.160
KSS function score preop	61.1 ± 7.4	64.1 ± 3.0	60.3 ± 6.1	55.0 ± 12.4	*p* < 0.05
KSS function score postop	77.3 ± 12.2	78.5 ± 9.7	79.3 ± 10.0	70.9 ± 18.5	0.395
Δ KSS function	16.3 ± 9.1	14.5 ± 9.5	19.0 ± 7.3	15.9 ± 10.5	*p* < 0.05
Recurrence rate (95% CI)	22.9% (15.1–34.9%)	11.4% (4.5–28.7%)	28% (14.9–52.5%)	42.9% (23.4–78.5%)	*p* < 0.05
Revision rate (95% CI)	14.9% (8.6–25.6%)	8.6% (2.9–25.3%)	12% (4.2–34.7%)	35.7% (17.7–72.1%)	*p* < 0.05

*Abbreviations*: *PI* peripatellar impingement, *AF* arthrofibrosis, *GS* generalized synovitis, *ROM* range of motion, *KSS* Knee Society Score, *CI* confidence interval

### Preoperative work up and diagnosis

Extensive efforts had been made to establish the aetiology preoperatively. Characteristics of pain, range of motion (ROM) limitation and joint stability were evaluated thoroughly. Erythrocyte sedimentation rate (ESR) and C-reactive protein (CRP) levels were used to screen for PJI. Radiographs including X-rays and computed tomography (CT) were used to assess possible component loosening, fracture, malposition, obvious malalignment and PE wear when revision was needed. A preoperative diagnosis was made whenever possible, mainly according to the diagnosis key points summarized in Table 2.

**Table 2:** Diagnostic efficacy of arthroscopic assessment according to indications

Indications	PI	AF	GS
Preop diagnosis key points	1. Crepitus and anterior knee pain (AKP) 2. Locking noted with increasing extension at approximately 40° of flexion 3. Resolved with a popping concussion with further extension.	1. Pain and limited ROM (< 90° flexion).	1. Recurrent painful effusion and/or occasional catching. 2. Slightly limited ROM and scattered peripatellar tenderness 3. Joint effusion signs in X-ray and CT.
Intraop diagnosis key points and procedures	1. Dense, well-defined fibrous bands on the backside of quadriceps tendon, which is caught in the femoral box with increasing extension. 2. The impinging soft tissue debrided.	1. Dense adhesions and fibrous bands throughout the joint. 2. Adhesions and bands debrided, controlled arthrolysis and haemostasis.	1. Hypertrophic GS. 2. Occasional synovium entrapment and bleeding. 3. Occasional signs of polyethylene delamination. 4. Synovectomy and haemostasis.

*Abbreviations*: *PI* peripatellar impingement, *AF* arthrofibrosis, *GS* generalized synovitis, *ROM* range of motion, *AKP* anterior knee pain, *preop* preoperative, *intraop* intraoperative

### Surgical techniques and postoperative rehabilitation

Arthroscopy was performed by one surgeon. Routinely, the anterolateral portal was used as the viewing portal, and the anteromedial arthroscope was used as the instrumentation portal. An additional superolateral and/or superomedial portal was created when necessary. The suprapatellar pouch, extensor mechanisms, medial and lateral gutters, intercondylar notch and all components were visualized systematically to check for the existence of adhesions, hypertrophied synovium, loose bodies, PE abrasions, etc. Preoperative diagnosis was confirmed intraoperatively, unless no major pathologic findings were identified. We used motorized and mechanical instruments (CrossBlade Series Cutters, Stryker, Greenwood, CO) to remove intra-articular fibrous bands and cyclops, hypertrophied or entrapped synovium. Intraoperative findings and arthroscopic procedures are summarized in Table 2. When performing extensive synovectomy, we elaborately coagulated the haemorrhage utilizing a radiofrequency ablator (SERFAS Energy System, Stryker, Kalamazoo, MI). Attention was paid not to scratch the prosthesis. After the procedure, all portals were sutured. We placed a suction drain and removed it after 24 h in most cases. A continuous passive motion (CPM) machine was started soon after surgery in patients with AF and at first postoperative day in other indications. Thromboembolism prophylaxis was given postoperatively as soon as safely possible. Prophylactic intravenous antibiotics were routinely used for 24 h, with the first dose given a minimum of 5 min before tourniquet inflation. We adhered to strict aseptic technique during the procedure.

### Clinical assessment and statistical analysis

The Knee Society Score (KSS) was utilized to evaluate the knee and functional status [18] of the patients before arthroscopy and at the latest FU or just before revision (if needed). Patients were categorized according to preoperative clinical characteristics and intraoperative findings as different indication groups. Considering an alpha (type 1) error of 0.05, beta (type 2) error of 0.2, mean incidence of PI, AF and GS being 3.5 %[12], 1.3% [19] and 1 %[6], respectively, and *d* = 0.05, the final estimated sample size for each group was determined to be 52 (PI), 20 (AF) and 16 (GS), respectively. Descriptive statistics were summarized as the means and standard deviations (SDs) for quantitative variables and as counts and frequencies for categorical variables. We used the Shapiro-Wilk test to examine the distribution of the data and Levene’s test to examine the equality for variances. Demographic data and preoperative radiographic measures were compared using the Kruskal-Wallis test for ordinal data and Fisher’s exact test for proportions among groups. Postoperative measures were compared to baseline using paired *t* tests. The statistical significance was set to *p* < 0.05. In post hoc analysis, to account for multiple comparisons, a Bonferroni corrected *p* value of *p* < 0.017 was used [20]. Data were statistically analysed using the SPSS Statistics 26.0 software program (IBM Corp., Armonk NY, USA).

## Results

All patients underwent arthroscopic surgery without intraoperative complications. However, there was postoperative morbidity. One patient with AF suffered from acute myocardial infarction (AMI) and pulmonary infection, which was treated with percutaneous coronary intervention and antibacterial treatment, and finally recovered. Three patients (one in each group) developed a PJI at 12, 35 and 62 months after knee arthroscopy. The PJI rate was 4.1% (95% confidence interval (CI 1.3–12.3%), and the non-infectious complication rate was 1.4% (95% CI 0.2–9.5%) in this case series.

As for therapeutic efficacy, which is presented in Table 1, patients in all 3 groups had improvements in ROM (*p* < 0.05), KSS knee score (*p* < 0.05) and KSS function score (*p* < 0.05). Postoperatively, among the three groups, the ROM, improvement in ROM, and KSS knee score varied (*p* < 0.05); however, there were no significant differences in postoperative KSS function, KSS knee improvement and function improvement. Games-Howell post hoc analysis showed that the PI group had the greatest postoperative ROM and KSS knee score postoperatively (*p* < 0.017), and patients with AF acquired the greatest improvement in ROM postoperatively (*p* < 0.017). Specifically, all patients with AF experienced a preliminary improvement in ROM immediately after surgery, and 18 of them remained asymptomatic without obvious limitation in ROM. The other 7 individuals experienced deterioration of ROM and continued pain with time. Three patients had a revision TKA.

**Fig. 2:**
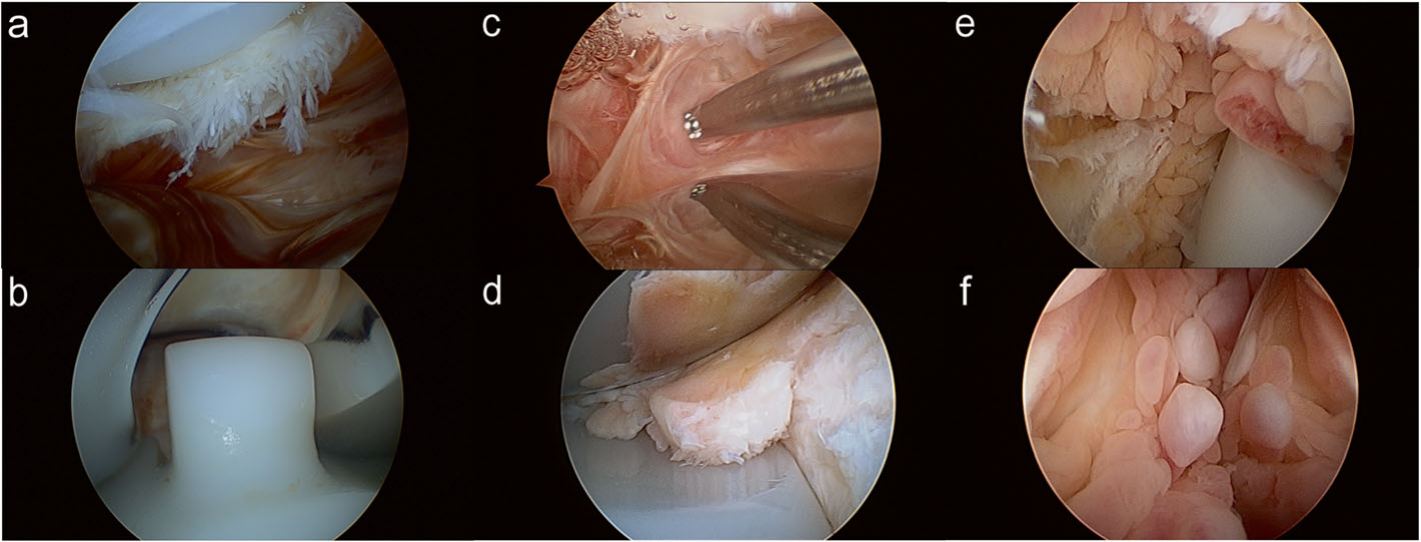
Typical intraoperative pictures of the lesions in PI, AF and GS. In patients with PI, the hypertrophied cyclops could be found at the backside of the quadriceps tendon which would contact with the distal edge of the trochlea, creating mechanical irritation. Signs of impingement could be seen (**a**). There were no hypertrophied synovium found in other areas of the joint (**b**). In patients with AF, hard scar tissue and fibrous bands could be found throughout the joint in the suprapatellar pouch (**c**), medial and lateral gutters and in the space between PE insert femoral prosthesis (**d**). All these scar tissue could prevent patients fully extend and flex their knees. In patients with GS, the hypertrophied and inflamed synovium could be detected throughout the whole joint in the medial and lateral gutters (**e**), suprapatellar pouch (**f**) and in the space between PE insert femoral prosthesis (**e**), which would lead to swelling of the knee joint. Signs of hyperemia in the synovium could be found, during extension and flexion, bleeding could occur due to entrapped synovium

Overall, the symptom recurrence rate was 22.9% (95% CI 15.1–34.9%), and the revision arthroplasty rate was 14.9% (95% CI 8.6–25.6%). There were significant differences on these 2 outcomes among the groups (*p* < 0.05, Fisher’s exact test). Post hoc analysis showed that the PI group had both the lowest symptom recurrence rate (11.4%, 95% CI 4.5–28.7%) and revision rate (8.6%, 95% CI 2.9–25.3%) (*p* < 0.017), while the GS group had both the highest recurrence rate (42.9%, 95% CI 23.4–78.5%) and revision rate (35.7%, 95% CI 17.7–72.1%) (*p* < 0.017). No patient underwent a repeat arthroscopy. One patient had a subsequent open arthrolysis but failed, and she had a revision afterwards. Typical intraoperative pictures of the lesions in PI, AF and GS were demonstrated in Fig. 2.

## Discussion

In the present study, we utilized arthroscopy in managing PI, GS and AF after TKA. The PJI rate was 4.1%, and the non-infectious complication rate was 1.4%. Most patients acquired remarkable improvements in ROM, KSS knee score and KSS function score, with a symptom recurrence rate of 23.0% and prosthesis revision rate of 14.9%. There were quite a few similar retrospective case series studies exploring the therapeutic efficacy of arthroscopy after arthroplasty whose main findings are summarized in Table 3. As shown in the table, the incidence of PJI ranged from 0–3.7%. Most studies with similar patient indications but a smaller sample size and shorter duration of FU reported that there were no post-arthroscopy infections [12–16]. We reported a higher infection rate of 4.1%. There were several hypotheses for the increased risk of infection as proposed by Werner et al., including violation of the joint capsule, persistent post-arthroscopy synovitis, bacterial seeding of the joint during KA and postoperative portal tracts as possible mechanisms for subsequent infection [22]. Lovro et al. studied 192 TKA patients who underwent subsequent knee arthroscopy using the information from the Medicare database with an FU of 5.45 years [17]. The incidence of revision for infection was 6.3%. The number (4.1%) in the current research was slightly lower than that (6.3%) in Lovro’s study. As with all database studies, Lovro’s research relied on ICD-9 and CPT codes, which were subject to inaccuracies. The authors used the code “infection”, which may possibly identify cases with infections other than PJI, which could potentially make the result falsely higher. Lovro et al. reported that the infection rate for post-arthroplasty patients without an arthroscopy was 2.1%. It was also higher than the majority of the results in other studies of the same era [23, 24]. For non-infectious complications, Heaven et al. reported in an SR that, across all 609 patients, only 1 intraoperative complication occurred—one patient had the arthroscopic instrumentation break in the knee, which was retrieved uneventfully. The overall complication rate was 0.5%, excluding those patients who received diagnostic and/or therapeutic arthroscopy for periprosthetic infection. We added one postoperative complication, and we warned that aggravation of comorbidities and life-threatening complications might occur, even after minimally invasive arthroscopic procedures.

**Table 3:** Summary of findings from previous studies

Author	Year	Study type	Indication	Sample size (knees)	Follow-up (months)	ROM	KSS knee score	KSS function score	Symptom recurrence rate	Revision rate	Infection rate	Non-infectious complications
Lucas et al. [12]	1999	Case series	Patellar clunk	30	12	105.6–106.1	64–93	65–89	3.3%	0	0	0
Koh et al. [7]	2008	Case series	Patellar clunk	12	13.4	NR	63.8–90.9	65.4–90.4	0	NR	NR	NR
Williams et al. [13]	1996	Case series	Arthrofibrosis	10	20	69.9–103	70.9–86.4	71–88	22.2%	22.2%	0	0
Mont et al. [8]	2006	Case series	Arthrofibrosis	18	30	63–94	34–77	NR	5.6%	0	NR	NR
Ohdera et al. [6]	2004	Case series	Hemarthrosis	6	24	NR	NR	NR	66.7%	NR	NR	NR
Kindsfater et al. [21]	1995	Case series	Hemarthrosis	4	NR	NR	NR	NR	50%	NR	NR	NR
Klinger et al. [10]	2005	Case series	Mixed	27	34	NR	71–85	69–83	33.3%	22.2%	1/27	0
van Mourik et al. [11]	1998	Case series	Mixed	27	NR	NR	NR	NR	41.7%	37.5%	NR	0
Wasilewski et al. [5]	1989	Case series	Mixed	12	25	33–67	NR	NR	NR	50%	NR	NR
Diduch et al. [14]	1997	Case series	Mixed	40	19.9	73–99*	87	85	27.5%	7.5%	0	0
Bocell et al. [15]	1991	Case series	Mixed	53	27	NR	NR	NR	7.5%	5.7%	0	0
Lovro et al. [17]	2020	Cohort study	Mixed	192	65.4	NR	NR	NR	NR	18.8%	6.3%	NR
Sekiya et al. [16]	2017	Case series	Mixed	30	36	116.7–117.4	75.1–86.9	NR	37%	0	0	0

*Abbreviation*: *NR* not reported

*The data only applies to patients with arthrofibrosis

For efficacy, Klinger et al. reported in a case series with similar patient indications that the average KSS knee score increased from 71 before arthroscopy to 85 postoperatively with an FU of 34 months, while the KSS function score increased from 69 to 83 patients [10]. In the current case series, patients gained comparable improvements in both KSS knee and function scores. For symptom recurrence, Heaven et al. reported that in an SR with approximately 2 years FU on average, of the 488 patients who underwent arthroscopy for therapeutic (not diagnostic) purposes, 85 (17.4%) patients experienced symptom recurrence after surgery [4]. In the current study, we presented a slightly higher recurrence rate of 23.0%. We think that the longer FU could potentially be the reason for this increased rate. In the SR, a more detailed statistical analysis of the pooled data was not possible because of different outcome measures used in different patient subgroups. In the current study, we found that patients with PI had the best results, in terms of lower recurrence and revision rates, while patients with GS had the highest risks of failure and prosthesis revision. Regarding the revision rate, in the current study, it was 14.9%. In the SR by Heaven et al., 19.7% of patients went on to require further operation, possibly including prosthesis revision and open arthrolysis [4]. The possible reason for the gap is that we did not include cases in which arthroscopy was applied for diagnostic purposes, whose intraoperative findings during arthroscopic evaluation may be tibial loosening, femoral prosthesis debonding and PJI. Revision surgeries or open debridement are often needed for these cases.

Some earlier studies involved patients with other indications, for example, PE wear, apparent PF maltracking, PJI, etc. In the current study, these patients were not studied and analysed, as severe PE wear was occurring less frequently [25] and a consensus had been reached regarding the standard management of PJI and apparent patella malalignment, for which prosthesis revision is the gold standard of treatment. We also did not include patients who underwent arthroscopy for diagnostic purposes, as there was very high heterogeneity among the subgroups of these patients. Additionally, the sample size for different subgroups was rather low (less than ten), which would undermine the statistical power [26]. From the clinical perspective, the application of arthroscopy as a diagnostic procedure should also be avoided in most cases, as it carries certain complication risks, and additionally, in 8 out of 19 patients who underwent arthroscopy for diagnostic purposes, no pathology could be identified. Meanwhile, recent literature has proposed new imaging tests (i.e. magnetic resonance (MR) imaging [22], MR angiography [27], synovial fluid cell phenotype analysis [28] and SPECT/CT [29]) to serve as appropriate diagnostic tools. These analyses were not utilized in the present study. However, they would be able to facilitate identifying the major aetiology and prevent unnecessary arthroscopic examinations.

**Fig. 3:**
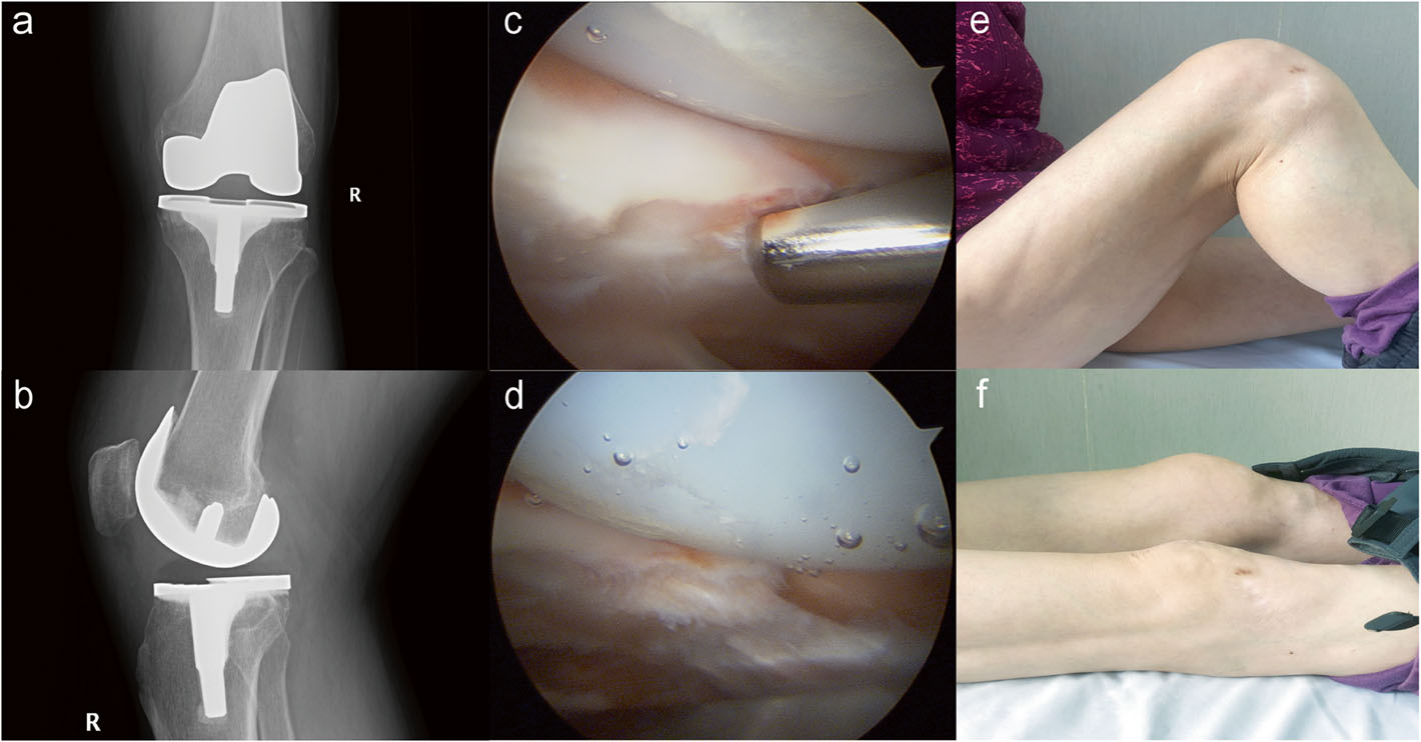
A typical case of PF impingement was presented. The patients suffered from painful crepitus during knee flexion of 40° after a CR TKA (**a**, **b**) and could not recover from conservative treatment. PJI, prosthesis loosening and joint instability had been ruled out prior to arthroscopy. During the procedure, the cyclops at the superior margin could be found (**c**) which was removed by arthroscopic shaver (**d**). The symptom relieved and patients gained a ROM of 105-0-0° postoperatively in 36 months follow-up (**e**, **f**)

There are some specific considerations on various patient groups based on our experience from this patient series. PI was not a rare complication [12] after TKA, especially in early PS design [30, 31]. Patients with PI usually presented with anterior knee pain (AKP), and additionally, crepitus could be found in history taking and/or physical exams, which could facilitate diagnosis. CT should be done to rule out obvious component malrotations, which could also lead to AKP. There were two types of PI, with the most common type being PF impingement. In this type, the hypertrophied cyclops and synovium at the backside of the quadriceps (shown in Fig. 2a) tendon have contact with the distal edge of the trochlea, creating mechanical irritation during knee extension. A typical case of PF impingement was presented in Fig. 3. Another type was tethered patella syndrome. Adhesion and fibrous bands could be found from the inferior pole of the patellar component to the intercondylar notch, tethering the patella inferiorly [15]. Intraoperatively, surgeons need to routinely check for the existence of hypertrophic fibrous bands, synovium and potential impingement around the patella. Mild patellar maltracking might occur simultaneously due to blocking of the cyclops or the tethering effect from the fibrous bands, and normal patellar motion has to be checked in the end. The conservative treatment often is unsatisfactory [30]. Lucas et al. [12] and Koh et al. [7] reported that most individuals with isolated PF impingement could expect an excellent result from arthroscopic resection, and the symptom recurrence rate was often low, ranging from 0–3.3% [7, 12, 15]. These results were consistent with the present study.

Patients with AF usually present with a stiff knee. In the present study, we define a stiff TKA as flexion under 90° and flexion contracture over 10°. Arthroscopy allows surgeons to release adhesions in a controlled manner. Williams et al. [15] and Mont et al. [16] reported an average improvement in ROM of approximately 30° after arthroscopic release [8, 13]. Our results were similar, with an improvement in ROM of 26.4°, and it should be noted that the improvements in the current study were generated from not only arthroscopic arthrolysis but also manipulation under anaesthesia (MUA) and intensive postoperative rehabilitation. Even though detectable clinical or radiographic abnormalities had already been ruled out, seven of twenty-five patients encountered a recurrence in the limitation of ROM; three of them underwent a prosthesis revision. Frustratingly, we were unable to figure out risk factors for treatment failure due to a relatively small sample size; thus, the therapeutic efficacy for AF is less predictable. According to the literature, arthroscopy might be helpful when the stiffness is caused by a tight posterior cruciate ligament (PCL) [13] or adhesions in the superior pouch or the medial and lateral gutter due to poor rehabilitation [13, 32]. In contrast, an extension deficit caused by a tight posterior capsule is not an ideal indication. We suggest a delicate analysis of potential aetiologies. If apparent surgical errors are present, arthroscopy should be avoided.

In the current series, patients with GS after TKA usually presented with recurrent swelling and/or hemarthrosis. According to the literature, the aetiology is also multifactorial. Systemic factors include anticoagulant use or presence of a bleeding disorder. Local factors include trauma, inflamed synovium or vascular anomaly (e.g. arteriovenous malformation) and injury. Cases caused by iatrogenic vascular injury usually present with swelling within 6 months postoperatively [33]. There were no such cases in this study. A more common mechanism and pathologic entity originates from the entrapment of hypertrophic synovium (shown in Fig. 2e). Varied conditions could cause synovium inflammation and hypertrophy (shown in Fig. 2f) (i.e. malaligned implants could lead to asymmetric PE wear and particle generation, causing synovial proliferation, subsequent impingement and bleeding). This is a more chronic process, usually occurring more than 1 year after TKA [33]. In the current study, all patients with GS belonged to this pathologic entity, and in four patients, signs of PE wear were detected. Conservative management consists of immobilization, cryotherapy, cessation of anticoagulants, and rest (and/or aspiration). However, only 30% of patients had resolution [21]. Angiography and selective embolization offers another choice, with the advantages of low infection risk, ability to be performed under local anaesthesia and quick rehabilitation postoperatively [34]. It was reported to be effective in more than 90% of cases [35]. However, repeat embolization may be necessary [36]. In patients with contraindications to angiography, arthroscopic synovectomy could be attempted, but its success is less predictable [6, 21, 37, 38]. Ohdera et al. [6] and Kindsfater et al. [21] reported a synovitis recurrence rate of 67% and 50%, respectively. Additionally, the pathologic site was not always identified arthroscopically. These failure rates and intraoperative findings were in line with those in the current study. Additionally, the angiographic evidence of contrast “blush”, indicating the pathologic site, is arthroscopically inaccessible sometimes (i.e. at the posterior capsule) [34]. In such circumstances, an open synovectomy is indicated, with a reported resolution rate of more than 90% [21, 39]. However, infection risk, wound complications, and prolonged rehabilitation would be clinical concerns. Revision is needed when obvious clinical or radiographic abnormalities are present. According to the results of the present study and existing literature, arthroscopic synovectomy should not be the first-line treatment for GS.

Several limitations should be acknowledged when drawing conclusions. The most important limitations include the retrospective observational study design and the absence of a control group, which can lead to potential recall bias. The absence of a control group probably results in an overestimation of the specific treatment effects, as the contextual effects contributing to the overall treatment effect cannot be determined [40], so the primary outcome in this study was the feasibility but not the therapeutic efficacy of arthroscopy after TKA. Second, the study design could also induce selection bias. Third, the sample size in the PI and GS groups did not reach the number derived from the sample size calculations due to the particular patient population presenting to a single surgeon in a single centre. The lost to follow-up rate was 19.6%, which poses a potential threat to the validity of the study [41]. However, the sample size in the current study was relatively large and the follow-up duration was comparatively long. In the current study, we provided information and evidence on the safety and therapeutic efficacy of arthroscopic debridement to manage PI, AF and GS after TKA.

## Conclusions

In the current study, we utilized arthroscopy to manage PI, AF and GS. With a longer FU and larger sample size, we reported rates of complication, PJI, symptom recurrence and prosthesis revision, as well as improvements in ROM and KSS in a more detailed manner in 3 different patient subgroups separately. Based on the result of the current study, arthroscopy after TKA is minimally invasive but not risk free. Avoiding unnecessary arthroscopic interventions in cases with clear technical errors is also important. Overall, in patients without obvious clinical and radiographic abnormalities, arthroscopy was feasible in managing a painful TKA in approximately 80% of cases, with improved ROM and KSS. Specifically, patients with PI typically had an excellent outcome. In most patients with AF, simultaneous MUA, early initiation of CPM and an aggressive rehabilitation process should be combined, and a profound recovery of ROM and joint function can be expected. In patients with GS, arthroscopic procedures should be limited and mainly applied when conservative treatment and selective embolization fail and the identified pathologic site is accessible arthroscopically.

## Data Availability

All of the data will be available for secondary analysis in necessary cases from the corresponding author through email address.
